# The Adsorption and Sensing Performances of Ir-modified MoS_2_ Monolayer toward SF_6_ Decomposition Products: A DFT Study

**DOI:** 10.3390/nano11010100

**Published:** 2021-01-04

**Authors:** Hongcheng Liu, Feipeng Wang, Kelin Hu, Tao Li, Yuyang Yan, Jian Li

**Affiliations:** State Key Laboratory of Power Transmission Equipment & System Security and New Technology, Chongqing University, Chongqing 400044, China; swulhcx@163.com (H.L.); 20140802026@cqu.edu.cn (K.H.); litao199811@outlook.com (T.L.); 20143232@cqu.edu.cn (Y.Y.)

**Keywords:** Ir-modified MoS_2_, decomposition components of SF_6_, adsorption and sensing, DFT

## Abstract

In this paper, the Ir-modified MoS_2_ monolayer is suggested as a novel gas sensor alternative for detecting the characteristic decomposition products of SF_6_, including H_2_S, SO_2_, and SOF_2_. The corresponding adsorption properties and sensing behaviors were systematically studied using the density functional theory (DFT) method. The theoretical calculation indicates that Ir modification can enhance the surface activity and improve the conductivity of the intrinsic MoS_2_. The physical structure formation, the density of states (DOS), deformation charge density (DCD), molecular orbital theory analysis, and work function (WF) were used to reveal the gas adsorption and sensing mechanism. These analyses demonstrated that the Ir-modified MoS_2_ monolayer used as sensing material displays high sensitivity to the target gases, especially for H_2_S gas. The gas sensitivity order and the recovery time of the sensing material to decomposition products were reasonably predicted. This contribution indicates the theoretical possibility of developing Ir-modified MoS_2_ as a gas sensor to detect characteristic decomposition gases of SF_6_.

## 1. Introduction

Due to the excellent insulation and arc-extinguishing ability of SF_6_ gas, it has been widely used in gas-insulated switchgear (GIS) [1,2]. During long-term operations, SF_6_ in GIS decomposes into different characteristic gases, such as H_2_S, SO_2_, SOF_2_, etc., when discharge faults occur [3,4]. Research showed that detecting these characteristic decomposition gases can reflect the operating status and potential faults of the gas-insulated equipment [5,6,7,8]. Therefore, the accurate detection of these gases is of important for the online monitoring of GIS and the safe operation of equipment. 

Among various detection methods, the metal-oxides-based gas sensor was considered a convenient and effective approach [9]. However, these traditional gas sensors have defects, such as low sensitivity and instability, limiting their further development [10,11]. Two-dimensional (2D) materials are widely used in gas sensing, catalysis, energy storage, etc., due to their large specific surface areas and distinctive physical and chemical properties [12,13,14]. The MoS_2_ monolayer was studied and reported as a potential gas-sensing material [15]. Rathi et al. studied the gas-sensing performance of La-MoS_2_ hybrid-heterostructure-based sensor of NO_2_ gas. They found that the sensing response of the La-decorated MoS_2_ gas sensor was six times more than the pristine MoS_2_, indicating that the fabricated sensor was suitable for NO_2_ detection [16]. Urs et al. reported the sensitivity of MoS_2_ modified with alloy nanoparticles to H_2_ at temperatures in the range of 30 to 100 °C. The results showed that the composite nanomaterials can enhance the response to H_2_, and this phenomenon was explained using the density functional theory (DFT) simulation [17]. Based on these studies, we deduced that the gas sensitivity of intrinsic MoS_2_ can be further effectively improved by introducing appropriate dopants, such as transition metal atoms or metal oxides [18]. In particular, iridium (Ir), which has excellent physicochemical stability, has been proven to effectively improve the gas sensitivity of 2D materials [19,20]. Thus, we speculated that the reasonable combination of Ir and MoS_2_ has potential for gas sensing. Considering the huge challenges faced by current sensor development and the importance of detecting SF_6_ decomposition components, the adsorption and sensing performance of Ir-modified MoS_2_ for these gases should be studied based on DFT for guiding the experiments.

In this paper, we predict the adsorption and sensing properties of Ir-modified MoS_2_ monolayer to SF_6_ decomposition components (H_2_S, SO_2,_ and SOF_2_) based on the DFT calculation. The most stable adsorption structure of Ir-modified MoS_2_ for different gases is proposed, and its electronic properties are also systematically explored. The gas-sensing response of the Ir-modified MoS_2_ nanomaterial-based sensor to the target gases was reasonably predicted. These results indicated that the Ir-modified MoS_2_-based gas sensor is a promising alternative for detecting the decomposition components of SF_6_.

## 2. Computational Detail

In this work, the DMol^3^ package based on the DFT method was used to study the electronic and adsorption characteristics [21]. The 4 × 4 MoS_2_ monolayer with the lattice constants of 12.664 × 12.664 Å was established, and its size proved to be large enough for gas adsorption [22]. To avoid influence between the adjacent layers of MoS_2_, we set the vacuum thickness to 20 Å. The electron exchange and correlation energy were treated by the generalized gradient approximation (GGA), Perdew–Burke–Ernzerhof (PBE), and the DFT-D method. The double numerical plus polarization (DNP) basis set was applied to deal with the relativistic effect of the molecular structure [23]. The k-point was set as 5 × 5 × 1 Monkhorst. To ensure the reasonableness and accuracy of the calculation, the energy convergence accuracy, maximum force, and maximum displacement in this study were selected as 10^−5^ Ha, 0.002 Ha/Å, and 0.005 Å, respectively [24]. The adsorption energy (*E_ad_*), charge transfer (*Q_t_*), energy gap (*E_g_*), and recovery time (*T_r_*) for each system were analyzed using the following equations:

*E_ad_* = *E_gas/Ir-modified MoS2_* − *E_gas_* − *E_Ir-modified MoS2_* (1)

*Q_t_* = *Q_a_* − *Q_b_* (2)

*E_g_* = |*E_LUMO_* − *E_HOMO_*| (3)

*T_r_* = *F*_0_^−1^exp(−*E_ad_*/*K_B_T_w_*) (4)

where *E_gas/Ir-modified MoS2_*, *E_gas_*_,_ and *E_Ir-modified MoS2_* denote the energy of the Ir-modified MoS_2_ structure adsorbed gas, the bare Ir-modified MoS_2_ substrate, and the isolated gas, respectively; *Q_a_* and *Q_b_* in represent the charge of gas molecules after and before adsorption, respectively. To study the conductivity change of various adsorption structures, we defined the energy gap (*E_g_*) depicted in Equation (3). HOMO and LUMO represent the highest occupied molecular orbital and the lowest occupied molecular orbital, respectively; the lower the *E_g_*, the higher the conductivity [25]. *T_r_*, *F*_0_ (10^12^ s^−1^), *K_B_* (8.62 × 10^−5^ eV K^−1^), and *T_w_* represent recovery time, attempt frequency, Boltzmann constant, and working temperature, respectively [26].

## 3. Results and Discussions

### 3.1. Structure and Properties of the Ir-modified MoS_2_ Monolayer

**Figure 1:**
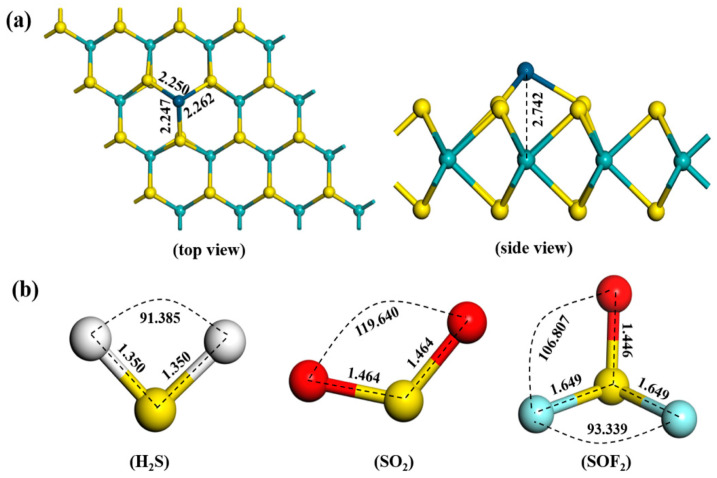
Structures of (**a**) Ir-modified MoS_2_ and (**b**) gas molecules.

The optimized structures of Ir-modified MoS_2_ and target gases are shown in Figure 1. Figure 1a illustrates that the Ir atom forms an optimal configuration with MoS_2_ at a distance of 2.742 Å directly above the Mo atom. This doping mode (the S vacancy) proves to be the most stable based on the formation energy analysis [27]. The three optimized Ir-S bond lengths of the Ir-modified MoS_2_ structure are 2.250, 2.262, and 2.247 Å. Figure 1b depicts the optimized configurations of three characteristic decomposition components of SF_6_. The bond length and bond angle of each gas molecule are marked in the figure, which is consistent with the literature [28,29].

**Figure 2:**
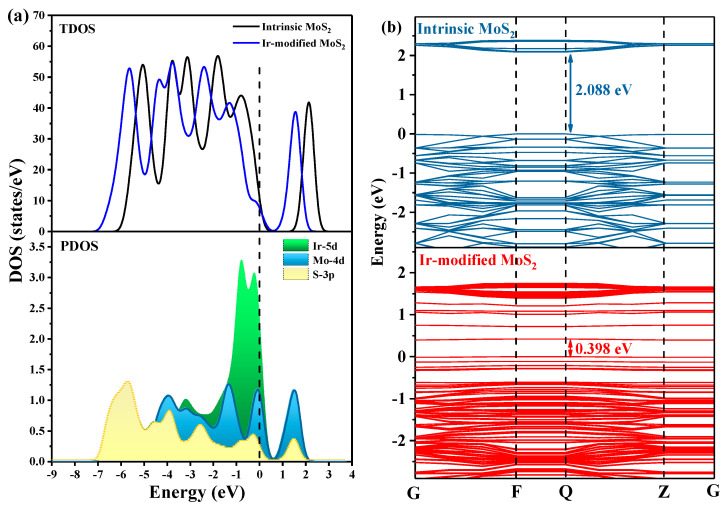
The (**a**) DOS distributions and (**b**) band structure of the intrinsic and Ir-modified MoS_2_ systems.

To further analyze the electronic properties of Ir-modified MoS_2_, the density of states (DOS) distributions and band structure are depicted in Figure 2. Figure 2a compares the total density of states (TDOS) of Ir-modified MoS_2_ shifted to the left with the intrinsic MoS_2_. This phenomenon is mainly attributed to the hybridization of the orbitals of introduced Ir atom with the orbitals of Mo and S atoms in MoS_2_. Specifically, the Ir-5d orbital overlaps with Mo-4d and S-3p orbitals in the range of −7.0 to 2.0 eV as observed in the distribution of partial density of states (PDOS), which indicated that the structure formed by the introduced Ir atom and MoS_2_ is stable. The Ir-5d peak appears near the Fermi level, indicating that electrons are more easily transferred to the conduction band after Ir atom was introduced, and the conductivity of the gas-sensing material increases. Figure 2b depicts the band structures of intrinsic and Ir-modified MoS_2_. We noticed that the bandgap of the composite system reduced from 2.088 to 0.398 eV after introducing an Ir atom due to the introduction of impurity levels near the Fermi level after doping with Ir atom. The electrons are more easily excited from the valence band to the conduction band.

**Figure 3:**
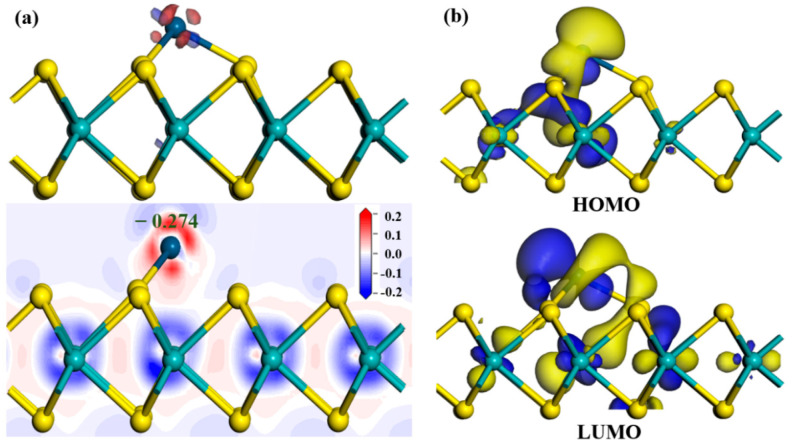
The (**a**) DCD, (**b**) HOMO, and LUMO distributions of Ir-modified MoS_2_ system.

Figure 3a displays the deformation charge density (DCD) of Ir-modified MoS_2_ monolayer. The red and blue regions in the figure represent electron accumulation and depletion, respectively [22]. The electrons are mainly concentrated around the Ir atom, which illustrates that the bonds formed by Ir atom and surrounding S atoms have a strong binding force [30]. The Ir atom acts as an electron acceptor and obtains 0.274 e from MoS_2_ monolayer, and the S mainly behaves as an electron donator. The HOMO and LUMO distributions of the Ir-modified MoS_2_ system are depicted in Figure 3b. We found that a large part of HOMO is distributed near the Ir atom, which indicates that the addition of an Ir atom provides more active sites on the surface of MoS_2_ and enhances the sensitivity of the material.

### 3.2. Gas Molecules Adsorption on the Ir-modified MoS_2_ Surface

#### 3.2.1. Structures of Different Adsorption Systems

**Figure 4:**
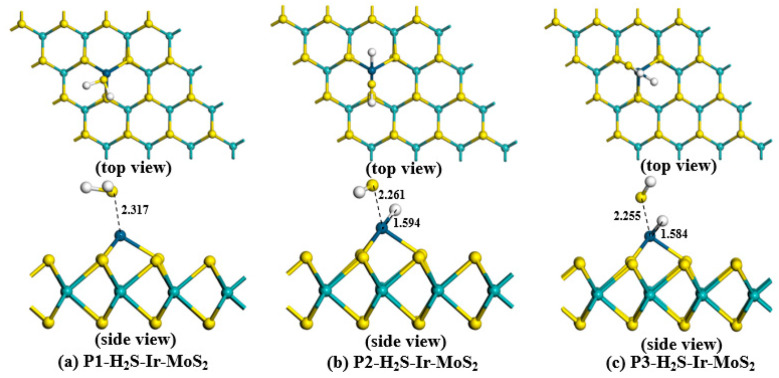
Various optimized configurations of H_2_S-Ir-MoS_2_ adsorption systems.

**Figure 5:**
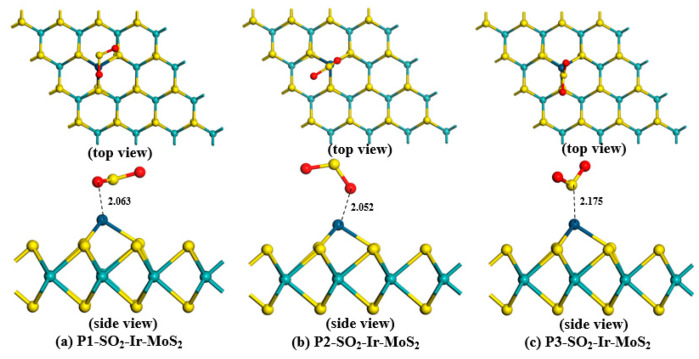
Different optimized structures of SO_2_ adsorption on the Ir-modified MoS_2_ surface.

**Figure 6:**
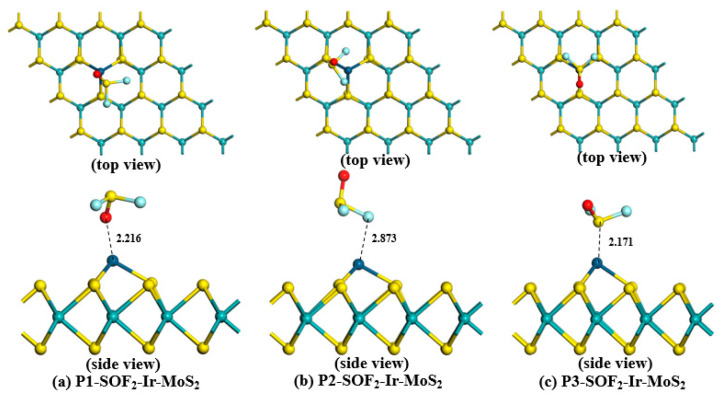
Adsorption systems of SOF_2_-Ir-MoS_2_ in different positions.

To obtain the most stable adsorption structure for each gas, we established various adsorption models in which the gases were close to the Ir-modified MoS_2_ surface at different positions. The optimized H_2_S-Ir-MoS_2_, SO_2_-Ir-dopd-MoS_2,_ and SOF_2_-Ir-dopd-MoS_2_ structures in various positions are depicted in Figure 4, Figure 5 and Figure 6, respectively. Based on the figures, we observed that all gas molecules were adsorbed on the surface of the Ir-modified MoS_2_ surface in different spatial positions. In particular, in the H_2_S adsorption systems of P2 and P3 depicted in Figure 4b,c, the H–S bond in the H_2_S gas broke, and the H atom formed a new chemical bond with the Ir atom, indicating that the adsorption of H_2_S gas is a chemical adsorption process. The adsorption parameters of Ir-modified MoS_2_ for various gases in different positions are depicted in Table 1.We observed from Table 1 that all the adsorption energies were negative, indicating that the adsorption of the target gases is an exothermic process. The absolute value of *E_ad_* in all systems was the largest for P3 structure, indicating that the adsorption structure is the most stable compared to the other two configurations, and the gas is most likely to be adsorbed on the surface of the material in this state during the adsorption reaction. The gas in each system was stably adsorbed on the Ir-modified MoS_2_ surface at different distances, and the specific adsorption distance values are listed in Table 1. For *Q_t_*, we found that the amount of charge transfer between H_2_S and the sensing material was the largest compared with SO_2_ and SOF_2_ gas adsorption systems, which illustrates that the H_2_S gas causes a greater change in the conductivity of the material during the adsorption process.

**Figure 7:**
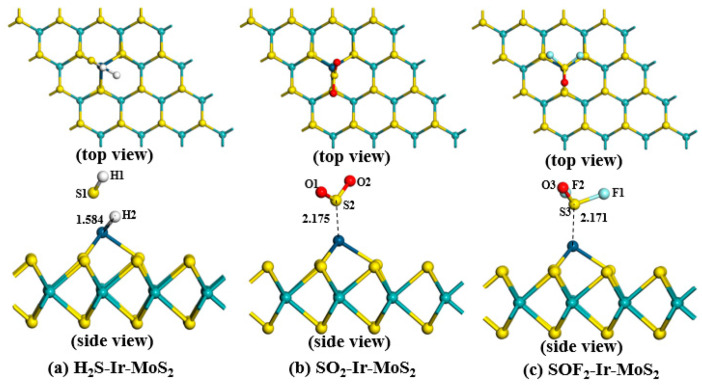
The most stable structures of SF_6_ decomposition components adsorbed on the Ir-modified MoS_2_ monolayer.

To further compare and analyze the different characteristics of Ir-modified MoS_2_ sensing material for different SF_6_ decomposition components, we selected the most stable structures (Position 3) for each gas adsorption system. The optimized configurations and adsorption parameters of target gases adsorbed on the Ir-modified MoS_2_ monolayer are depicted in Figure 7 and Table 2, respectively. We suggest, based on Figure 7a, that the S–H bond in H_2_S gas was broken due to the strong metallicity of Ir atom during the adsorption process of H_2_S gas, which led to the formation of a new H2–Ir bond between H2 atom and Ir atom with a bond length of 1.584 Å. The H1–S1 bond length is 1.357 Å, which is slightly elongated compared to the bond length of the gas before adsorption (1.350 Å). For the SO_2_-Ir-MoS_2_ and SOF_2_-Ir-MoS_2_ adsorption systems depicted in Figure 7b,c, respectively, we observed that the S atom in the gas molecules is close to the Ir atom to form the stable structures. The bond lengths of S2–O1 and S2–O2 in SO_2_ gas are 1.467 and 1.468 Å, respectively. The bond lengths of S3–O3, S3–F2, and S3–F1 in SOF_2_ gas are 1.449, 1.642, and 1.643 Å, respectively. All of the bond lengths of SO_2_ and SOF_2_ gases undergo small changes during the adsorption progress, suggesting that gases have interactions with the sensing material.

Adsorption systems are displayed in Table 2. The *E_ad_* of H_2_S-Ir-MoS_2_ was calculated to be −2.323 eV, which is smaller than the SO_2_-Ir-MoS_2_ (−1.757 eV) and SOF_2_-Ir-MoS_2_ (−1.492 eV) systems. This result suggested that all adsorption reactions can proceed spontaneously, the adsorption process of the H_2_S gas is the strongest, and the formed structure is the most stable among the three systems. According to the definition of adsorption distance, the *D* of H_2_S-Ir-MoS_2_, SO_2_-Ir-MoS_2,_ and SOF_2_-Ir-MoS_2_ is 1.583, 2.175, and 2.171 Å, respectively. Based on the Mulliken population analysis, we calculated the *Q_t_* of the three adsorption systems as 0.286, 0.114, and 0.154 e for H_2_S-Ir-MoS_2_, SO_2_-Ir-MoS_2,_ and SOF_2_-Ir-MoS_2_ configurations, respectively. The largest *Q_t_* of H_2_S-Ir-MoS_2_ indicates that the H_2_S gas interacts strongly with the Ir-modified MoS_2_ system, which is consistent with the calculated results of adsorption energy.

#### 3.2.2. DOS Analysis of Different Adsorption Systems

**Figure 8:**
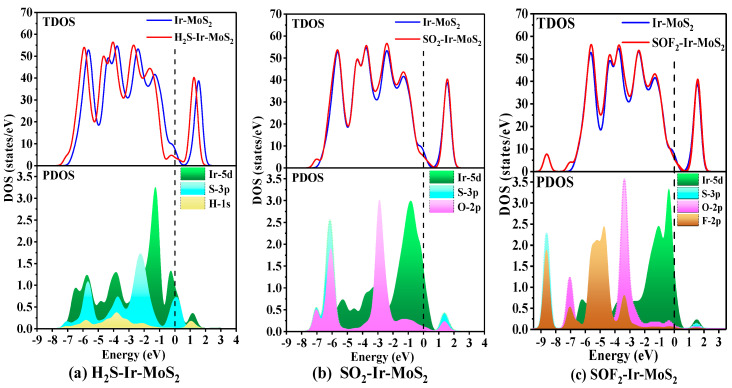
The DOS distributions of different adsorption systems.

The DOS distributions were used to study the electronic properties and gas sensitivity of SF_6_ decomposition components adsorbed on the Ir-modified MoS_2_ monolayer, and the results are depicted in Figure 8. Figure 8a depicts the overall TDOS of the H_2_S-Ir-MoS_2_ system moving to the left after adsorbing H_2_S gas, indicating that the electrons easily fill in the conduction band. The huge change in TDOS that happened near the Fermi level indicated that the Ir-modified MoS_2_ gas-sensing material has strong interactions with H_2_S gas, and the gas sensitivity is improved. The peaks of the PDOS spectrum overlap among Ir, S, and H atoms, suggesting that the adsorption structure has good stability. The hybridization of Ir-3p and S-3p orbitals should be responsible for the huge changes in TDOS near the Fermi level. In the SO_2_-Ir-MoS_2_ structure displayed in Figure 8b, the TDOS decreases around the Fermi level after SO_2_ gas adsorption. This phenomenon illustrates that the number of free electrons decreases after gas adsorption and the resistance of the material increases. We observed the PDOS distributions of Ir-5d orbital near the Fermi level, which may be the main reason for the decrease in TDOS. The hybridization of Ir-5d and S-3p orbitals increases the TDOS near the energy level of −7.0 eV after gas adsorption. For the SOF_2_-Ir-MoS_2_ system depicted in Figure 8c, the TDOS of the SOF_2_-Ir-MoS_2_ adsorption system slightly changes near the Fermi level compared with the DOS of Ir-MoS_2_, indicating that the material has weak gas-sensitivity to SOF_2_ gas. From the PDOS distributions, we found that the novel peaks appearing in DOS at the range of −9.0 to −8.0 eV are mainly caused by the hybridization of the S-3p and F-2p orbitals.

#### 3.2.3. DCD Analysis of Various Adsorption Systems

**Figure 9:**
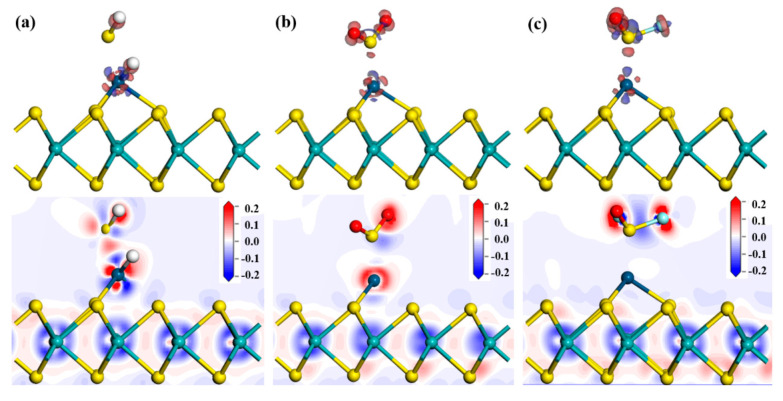
The DCD of (**a**) H_2_S-Ir-MoS_2_, (**b**) SO_2_-Ir-MoS_2,_ and (**c**) SOF_2_-Ir-MoS_2_ adsorption systems.

The interactions between SF_6_ decomposition components and Ir-modified sensitive materials were studied via the DCD analysis. Figure 9 depicts the corresponding DCD results of various adsorption systems. The charge accumulation in all structures is mainly concentrated on the gas molecules, and charge depletion is distributed around the Ir atom. This distribution suggests that the gases have interactions with the Ir-modified MoS_2_ material, which is also consistent with the DOS analysis results. In these adsorption systems, the SF_6_ decomposition components act as electron donors, while the Ir-modified MoS_2_ material acts as an electron acceptor. Compared with SO_2_-Ir-MoS_2_ and SOF_2_-Ir-MoS_2_ adsorption configurations, there is a large amount of charge accumulation and dissipation between the H_2_S gas and the Ir-modified MoS_2_ layer, which is caused by the strong reaction in the adsorption process [31].

#### 3.2.4. Frontier Molecular Orbital Analysis of Different Systems

**Figure 10:**
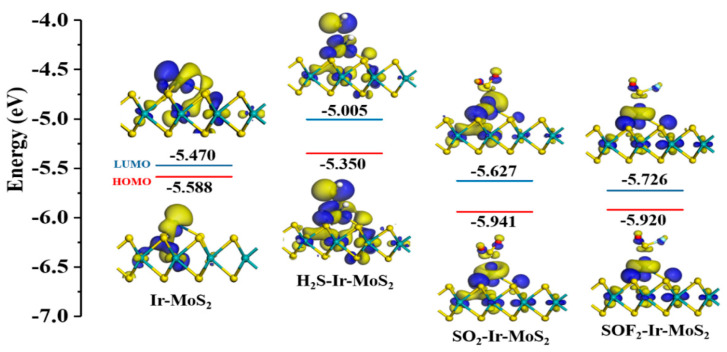
The HOMO and LUMO distributions of various systems.

The HOMO and LUMO distributions were used to study the electronic behavior of various adsorption systems. According to the frontier molecular orbital analysis, we analyzed the possible change trends of material conductivity to predict the gas-sensing performance of materials [32]. After the target gas is adsorbed, the electron clouds of Ir-modified MoS_2_ material is redistributed, causing changes in the energy values of HOMO and LUMO, as depicted in Figure 10. A large amount of HOMO is distributed on and around the gas, especially in the H_2_S-Ir-MoS_2_ system, indicating that these electrons are not bound and can undergo charge transfer during the adsorption reaction. The Ir-modified MoS_2_ sensitive material has obvious electron transfer behavior for the adsorption of these three target gases; thus, we speculate that Ir-modified MoS_2_ gas-sensing material can be used as a resistive gas sensor to detect H_2_S, SO_2,_ and SOF_2_ gases.

### 3.3. Gas-Sensing Prediction of Ir-modified MoS_2_ to SF_6_ Decomposition Products

**Figure 11:**
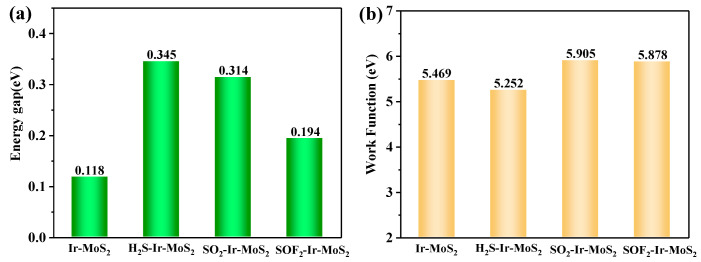
The (**a**) energy gap and (**b**) working function for different optimized systems.

After gas adsorption, a large change in *E_g_* indicates that the conductivity of the sensing material increases based on the definition of resistive gas sensor sensitivity [33]. The comparative analysis of *E_g_* for different optimized systems are proposed and displayed in Figure 11a. The *E_g_* increased to varying degrees after gas adsorption due to the different changes in the energy levels of HOMO and LUMO (marked in Figure 10). Compared with the *E_g_* of the Ir-modified MoS_2_, the degree of *E_g_* change in these systems is as follows: H_2_S-Ir-MoS_2_ > SO_2_-Ir-MoS_2_ > SOF_2_-Ir-MoS_2_. Combining the above results, we suggest that the gas sensitivity order of the Ir-modified MoS_2_ to these SF_6_ decomposition products is H_2_S > SO_2_ > SOF_2_. Work function (WF) refers to the minimum energy required for electrons to release from the surface. It represents the contact barrier between the target gas and the material during the gas adsorption process [31]. Figure 11b shows the calculated values of WF for various optimized structures. We observed that WF decreases to 5.252 eV after the adsorption of H_2_S gas, but increases to 5.905 and 5.878 eV for SO_2_ and SOF_2_, respectively, compared with the Ir-modified MoS_2_ system (5.469 eV). In other words, the smaller work function value of H_2_S-Ir-MoS_2_ indicates that H_2_S more easily adsorbs on the Ir-modified MoS_2_ material compared with the other two systems.

**Figure 12:**
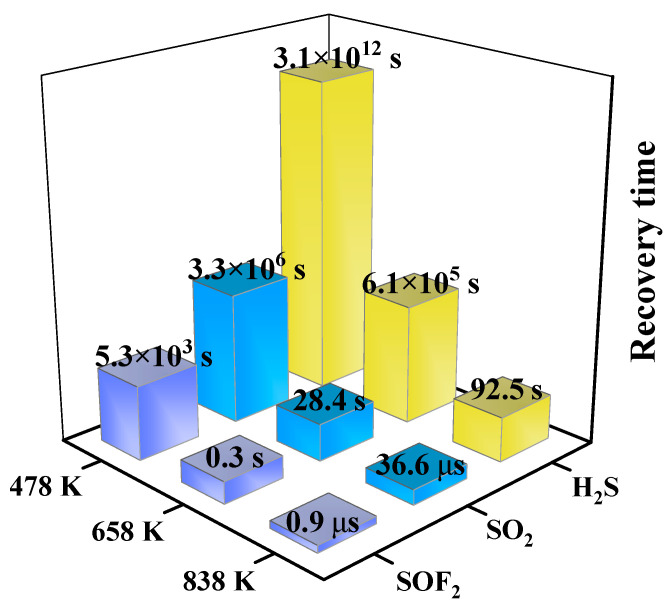
The predicted recovery time of various optimized systems.

The predicted recovery time for the Ir-modified MoS_2_ based sensor is displayed in Figure 12. The recovery time decreases with the increase in temperature due to the rapid desorption of gas molecules at high temperatures. The sequence of the recovery time for these gases at the same temperature is as follows: SOF_2_ < SO_2_ < H_2_S. Although the recovery time of H_2_S is very long due to the strong adsorption capacity, the time can be less than 2 min by appropriately increasing the working temperature during the experimental test. This result can provide a theoretical basis for guiding the gas-sensing performance test experiment.

## 4. Conclusions

In this work, we used the theoretical calculation based on the DFT method to investigate the adsorption characteristic and gas-sensing mechanism of Ir-modified MoS_2_ to decomposition components of SF_6_, including H_2_S, SO_2_, and SOF_2_. We optimized and analyzed the geometric parameters and electronic properties of the Ir-modified MoS_2_ system. The results indicated that the introduction of the Ir atom enhances the surface activity of the material and reduces the bandgap of intrinsic MoS_2_ from 2.088 to 0.398 eV as well as increases the conductivity. The most stable adsorption structure of Ir-modified MoS_2_ for different gases was proposed, and their electronic properties were systematically explored via analyzing the DOS, DCD, molecular orbital theory, and WF. The gas-sensing mechanism study demonstrated that Ir-modified MoS_2_ monolayer can adsorb the target gases and cause microscopic electron behavior, especially for H_2_S gas. The gas sensitivity order of SF_6_ decomposition products was predicted as follows: H_2_S > SO_2_ > SOF_2_. The predicted recovery time of the sensor to all target gases can be less than 2 min by appropriately increasing the temperature. Based on these results, the Ir-modified MoS_2_ is suggested as a potential candidate for detecting decomposition components of SF_6_.

## Figures and Tables

**Table 1:** The adsorption parameters of Ir-modified MoS_2_ for various gases in different positions.

System	Adsorption Position	*E_ad_* (eV)	*D* (Å)	*Q_t_* (e)
H_2_S-Ir-MoS_2_	Position 1 (Figure 4a)	−1.578	2.317	0.341
	Position 2 (Figure 4b)	−2.310	1.594	0.292
	Position 3 (Figure 4c)	−2.323	1.584	0.286
SO_2_-Ir-MoS_2_	Position 1 (Figure 5a)	−1.656	2.063	−0.158
	Position 2 (Figure 5b)	−1.053	2.052	−0.190
	Position 3 (Figure 5c)	−1.757	2.175	0.114
SOF_2_-Ir-MoS_2_	Position 1 (Figure 6a)	−0.411	2.216	0.069
	Position 2 (Figure 6b)	−0.104	2.873	0.048
	Position 3 (Figure 6c)	−1.492	2.171	0.154

**Table 2:** The parameters of different Ir-modified MoS_2_ adsorption systems.

System	*E_ad_* (eV)	*D* (Å)	*Q_t_* (e)
H_2_S-Ir-MoS_2_	−2.323	1.584	0.286
SO_2_-Ir-MoS_2_	−1.757	2.175	0.114
SOF_2_-Ir-MoS_2_	−1.492	2.171	0.154
